# Study on the spatial spillover effect of land use type change on carbon emissions

**DOI:** 10.1038/s41598-023-39383-0

**Published:** 2023-07-27

**Authors:** Ruiwu Zhang, Jun Ying, Yiqi Zhang

**Affiliations:** 1 College of Landscape Architecture and Landscape Architecture, Zhejiang A&F University, Hangzhou, 311300 Zhejiang China; 2 Institute of Ecological Civilization & Institute of Carbon Neutrality, Zhejiang A&F University, Hangzhou, 311300 Zhejiang China

**Keywords:** Climate change, Environmental sciences

## Abstract

Land use change affects the terrestrial carbon cycle, a crucial factor in attaining energy conservation and emission reduction under climate change. This study constructs panel data for thirteen Hangzhou districts and municipalities from 2000 to 2020. Using the spatial Durbin model, it analyzes the spatial spillover effect of land use change on carbon emissions. The results show that the spatial distribution of carbon emissions in Hangzhou continues to increase with positive spatial autocorrelation, and the spatial distribution shows "high-high" and "low-low" clustering. The expansion of construction land is the main reason for the increase in carbon emissions, and the inhibitory effect of water area on carbon emissions is more potent than woodland. The area of cultivated land and construction land has a positive spillover effect on carbon emissions, while the woodland area has a negative spillover effect on carbon emissions. To promote urban low-carbon development, maximizing the spatial spillover effect of land use and establishing a collaborative governance system between districts and counties is crucial.

## Introduction

Carbon emissions have contributed to the intensification of global climate change, threatening human survival and sustainable development. In response to the hazard posed by climate change, the Paris Agreement was convened in 2015 to limit global temperature rise to less than 2 °C above pre-industrial level^1^. According to the International Energy Agency^2^, global energy-related carbon emissions stabilized in 2019, which occurred against the backdrop of 2.9% global economic growth^3^. Developing carbon reduction strategies coordinated with economic growth is an aim shared by all nations in the context of climate change.

As a location for human activities, land carries economic activities and generates a large amount of energy consumption. Under the influence of human activities, the land undergoes dynamic changes that have varying consequences for humans and the environment^4,5^. Relevant studies have shown that carbon emissions from land use change are second only to fossil fuels and have risen steadily over the past several decades^6^. Variations in land use types engender uncertainties in land cover and carbon emission variations due to climate change^7^. To construct a synergistic governance system among regions, achieve sustainable land use, promote low-carbon urban economic development, and enhance the ecological environment, it is essential to analyze the relationship between land use and carbon emissions in terms of spatial relationships.

Many scholars have studied the relationship between land use and carbon emissions in recent decades. Using remote sensing satellites and dynamic models, scholars have analyzed the evolutionary characteristics of land use change and identified the expansion of built-up land into vegetated regions as the primary characteristic of global land use change; this shifting pattern has accelerated steadily over time^8–12^. According to a study by Barati et al.^13^, land use change results in land degradation, which destroys biodiversity and land complexity, thereby contributing to global climate change. Several researchers have investigated the carbon cycle to land use change. Wu et al.^14^ found that the carbon cycle has accelerated in recent years, and carbon dioxide fixed by plant photosynthesis is being lost to the atmosphere faster. Quesada et al.^15^ modeled the carbon cycle under future land use change and discovered that by the end of the twenty-first century, deforestation would reduce land carbon storage by about 24%. Gasser et al.^16^ assessed the carbon emissions from global land use change. They concluded that the loss of carbon stocks under the anthropogenic influence is causing a rapid increase in net carbon emissions. In addition, many studies on the relationship between land use type changes and carbon emissions have concluded that distinctions in the impact of various land use types on carbon emissions are attributable to varying levels of human economic activity. For example, population clustering, economic development, transportation, and factory enterprises in cities contribute to carbon emissions by increasing energy consumption^17–19^, carbon emissions from agricultural land come from tillage, farming, and over-fertilization^20,21^, and the rate of carbon emissions from the watershed environment under the influence of fishing, agriculture, production, and trade is greater than the rate of carbon sinks, making it an essential source of carbon^22,23^. Although these studies demonstrated the relationship between land use type changes and carbon emissions, they were limited to independent study areas. They did not account for the effects of land use type changes on carbon emissions in other regions based on the role of spatial relationships.

The spatial spillover effect is crucial when analyzing spatial relationships, which refers to changes in regional environmental elements on the same ecological aspects in the surrounding neighboring areas under the effect of inter-regional association. Although comparatively little attention has been paid to studying the spatial relationship between changes in land use type and carbon emissions, empirical tests have shown that the impact of land use changes on the carbon cycle among different regions has apparent spatial reciprocity^24,25^. The study of Kuschnig et al.^26^ found that the influence of economic production activities drives the encroachment of the surrounding neighboring areas on natural ecological land types, which Cary and Bekun^27^ and Leijten et al.^28^ found that high economic countries have strict environmental protection measures to avoid damage to the indigenous environment, thereby shifting the access to natural resources to the neighboring backward countries, resulting in an increase in the encroachment of natural ecological land types. This impact on interregional carbon stocks will further cause the spatial spillover effect of carbon emissions. Similarly, a study by Zeng et al.^29^ found that energy consumption by urban expansion has a spatial correlation effect among Chinese provinces. Liu and Liu^30^ found that the spatial correlation of energy consumption forms a spatial spillover effect of carbon emissions, causing the urbanization process to have varying degrees of impact on carbon emissions in neighboring regions. In the study of agricultural land, researchers discovered that economic and technological development prompted the interaction of agricultural activities between regions^20,31,32^, which not only promoted the expansion of agricultural development and the arable land area between regions^33^ but also had varying degrees of spatial spillover effects on agricultural carbon emissions^34,35^. Therefore, economy and energy are essential factors that affect the spatial effects of land use type changes on carbon emissions. However, in some studies, academics believe that economic investment will promote more energy consumption and carbon emissions in the early stages of economic development. However, when the economy develops to a certain extent, it will promote the progress of low-carbon production technology, thereby improving production efficiency and reducing carbon emissions. Therefore, the spatial effects of carbon emissions vary at different stages of development^17,19,36,37^.

In summary, changes in land use types reflect the direction of human activities and development, which alter the inter-regional carbon cycle and influence the neigh-boring areas' carbon cycle due to spatial correlation, resulting in changes in interregional carbon emissions. Therefore, this study constructs a spatial panel model for carbon emissions. Using panel data from 13 districts and municipalities in Hangzhou from 2000 to 2020, the influence of land use type on carbon emissions and its spatial spillover effect are analyzed. This study examines the interrelationship between land use types and carbon emissions in various Hangzhou regions. From the perspective of low-carbon urban development, it offers optimization suggestions for constructing a synergistic land use governance system among districts and counties.

## Materials and methods

### Study area

**Figure 1:**
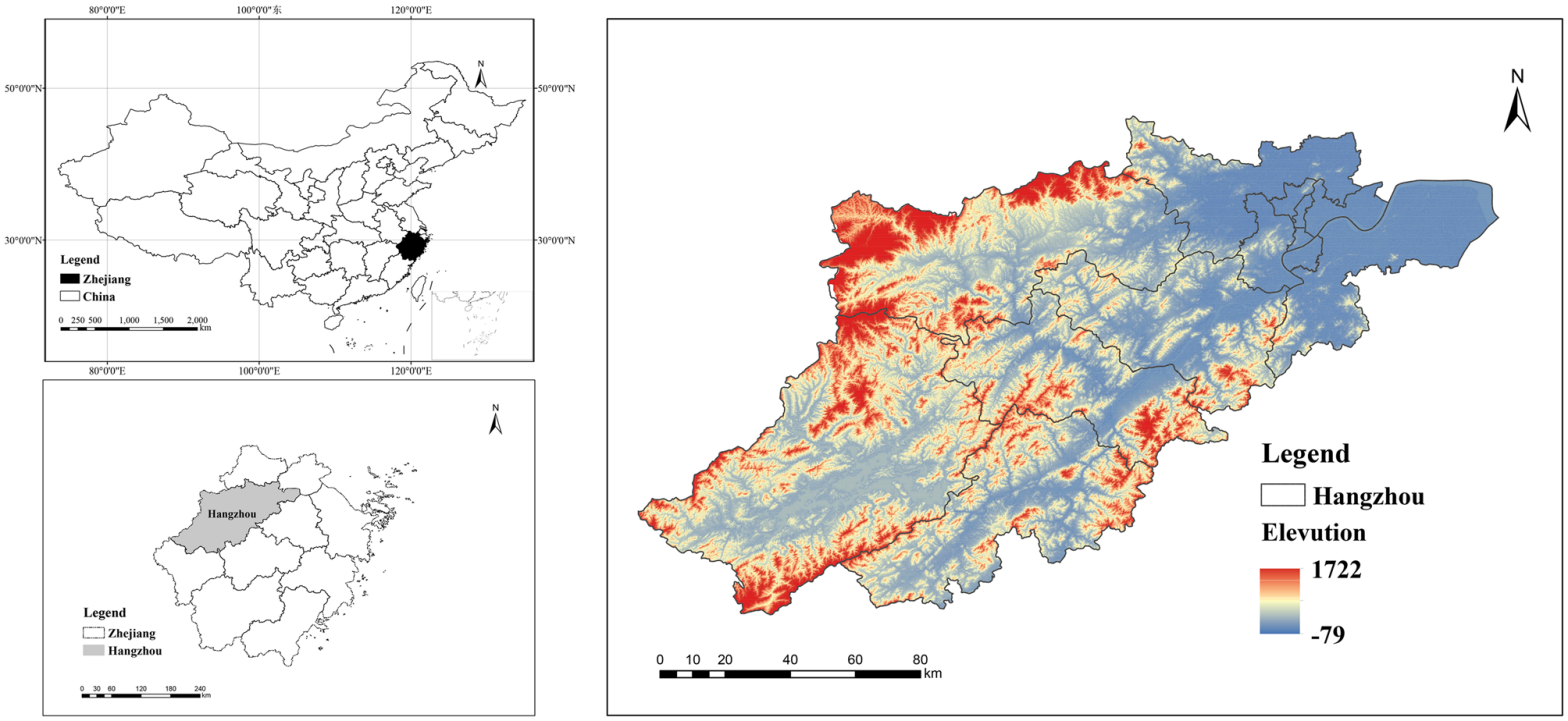
Location of the study area (using Arc GIS10.8. Source of vector maps: http://datav.aliyun.com/portal/school/atlas/area_selector. Source of DEM data: https://www.gscloud.cn/sources/?cdataid=302&pdataid=10).

This study used the study area of Hangzhou City in East China (Fig. 1). Hangzhou is located in the northern portion of Zhejiang Province at latitude 29.183–30.55 North and longitude 118.35–120.5 East. Its total land area is 16,596 km^2^. It consists of ten districts (Shangcheng District, Xiacheng District, Jianggan District, Gongshu District, Xihu District, Binjiang District, Xiaoshan District, Yuhang District, Lin'an District, and Fuyang District), two counties (Tonglu County and Chun'an County), and one city (Jiande City). As one of the most developed cities in eastern China, Hangzhou's population grew from 6,215,800 at the end of the year 2000 to 11,965,000 at the end of the year 2020, and its gross domestic product increased from 138.256 billion yuan to 1,610.583 billion yuan, placing it ninth among Chinese cities. According to the current status of land use, the arable land area of Hangzhou will account for approximately 17.21% of the city's land area in 2020, while woodland area will account for about 67.73%, grassland will account for about 2.33%, water area will account for approximately 5.22%, construction land will account for about 7.47%, and unused land will account for about 0.04%. Due to changes in land use patterns and an increase in energy demand, socioeconomic development continues to be a major cause of carbon emissions. To achieve carbon emission reduction goals, it is therefore of great practical importance to examine the relationship between land use type changes and carbon emissions in the context of analyzing the future socioeconomic development of the region.

### Data source

**Figure 2:**
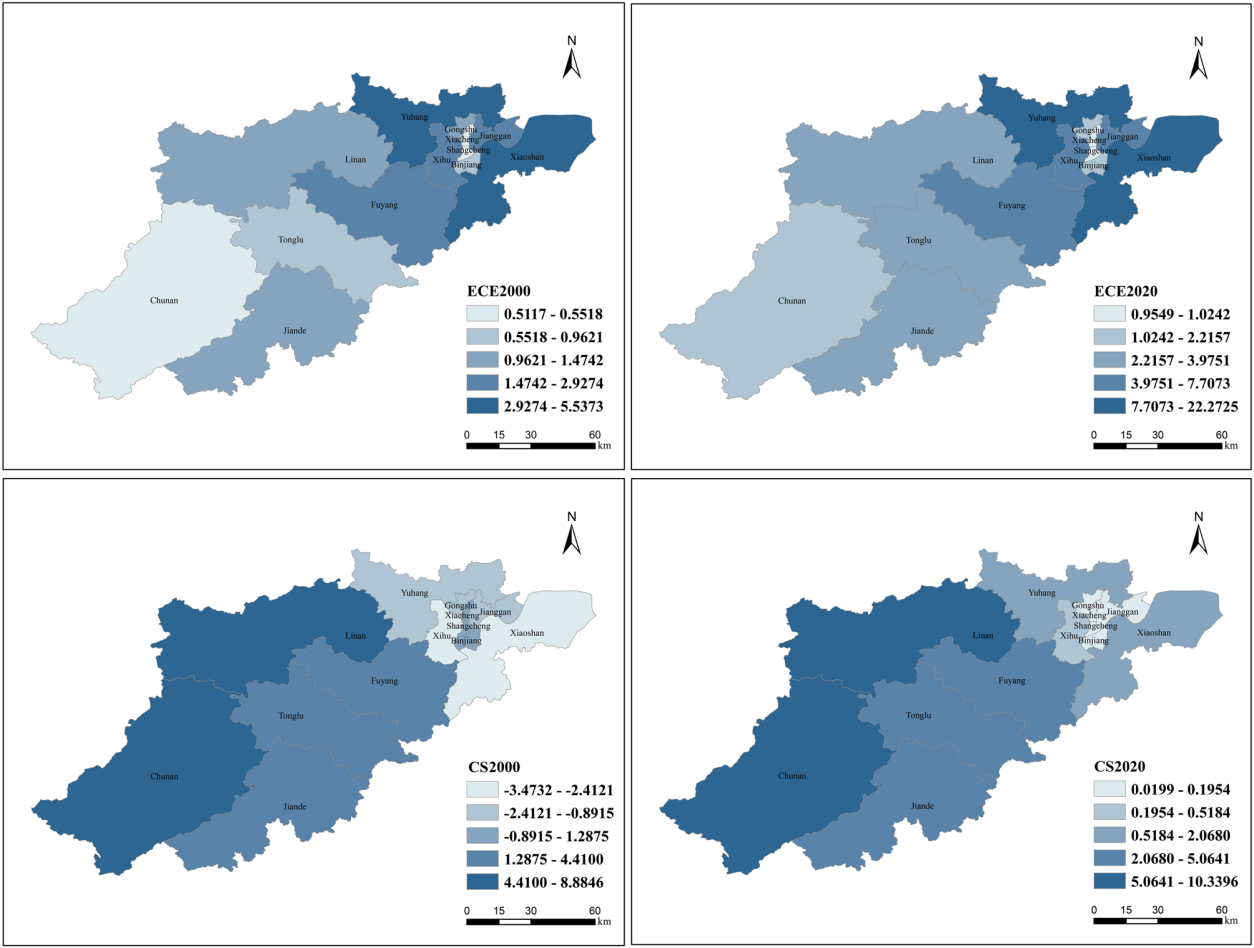
Spatial distribution of energy related carbon emissions and carbon storage in Hangzhou from 2000 to 2020 (using Arc GIS 10.8. Source of vector maps: http://datav.aliyun.com/portal/school/atlas/area_selector. Source of China's county-level carbon emissions and carbon storage data).

This research employed the following data: This research employed the following data: The 30 × 30 m resolution Chinese land use monitoring data (http://www.resdc.cn) includes six first-level classifications, such as construction land, cropland, woodland, and grassland. The carbon emission data is sourced from Chen et al.^38^ calculations at the county level in China, which include the amount of energy-related carbon sources and the carbon sequestration of natural ecosystems (Fig. 2). In their study, provincial carbon emissions were calculated using the consumption of 17 fossil fuels and each energy source's calorific value, carbon content, and carbon oxidation coefficient in order to determine energy-related carbon emissions. The total DN value of DMSP/OLS nighttime light image was then used to calculate carbon emissions at the county level. For calculating carbon storage, MOD17A3 products, land cover data, and county DN values are utilised. After testing, the R^2^ values of the two are 0.9595 and 0.9555, respectively, indicating that the availability of this data is high. Carbon storage (CS) is subtracted from energy-related carbon emissions (ECE) to determine Hangzhou's carbon emissions (CE).

### Analytical methods

In this study, we primarily employed the spatial autocorrelation test and the spatial economic model, where the spatial autocorrelation test incorporates both the global and local Moran'I tests. The global Moran's I test and the Spatial Econometric Model were analysed with Stata 17 software, while the local Moran'I test was analysed with GIS 10.8 software.

### Spatial autocorrelation test

Moran's I is the most frequently employed indicator for testing the spatial auto-correlation of variables, reflecting the degree of correlation between various regions of the measured variables, primarily comprising global spatial autocorrelation and local spatial autocorrelation^39^. The global autocorrelation is shown in Eq. (1):

1 $${\text{I}}_{{\text{g}}} = \frac{{\sum\nolimits_{{{\text{i}} = 1}}^{{\text{n}}} {\sum\nolimits_{{{\text{j}} = 1}}^{{\text{n}}} {{\text{W}}_{{{\text{ij}}}} \left( {{\text{x}}_{{\text{i}}} - {\overline{\text{x}}}} \right)\left( {{\text{x}}_{{\text{j}}} - {\overline{\text{x}}}} \right)} } }}{{{\text{S}}^{2} \sum\nolimits_{{{\text{i}} = 1}}^{{\text{n}}} {\sum\nolimits_{{{\text{j}} = 1}}^{{\text{n}}} {{\text{W}}_{{{\text{ij}}}} } } }}$$

The local Moran's I is shown in Eq. (2):

2 $${\text{I}}_{{\text{i}}} = \frac{{\left( {{\text{x}}_{{\text{i}}} - {\overline{\text{x}}}} \right)}}{{{\text{S}}^{2} }}\sum\limits_{{{\text{j}} = 1}}^{{\text{n}}} {{\text{W}}_{{{\text{ij}}}} } \left( {{\text{x}}_{{\text{j}}} - {\overline{\text{x}}}} \right)$$

where ${\mathrm{W}}_{\mathrm{ij}}$ is the spatial weight matrix, ${\mathrm{x}}_{\mathrm{i}}$ and ${\mathrm{x}}_{\mathrm{j}}$ are the carbon emissions of region i and region j, respectively, and ${\overline{\text{x}}}$ is the average value of regional carbon emissions. The Moran index takes a value between − 1 and 1. If the value is greater than 0, it means there is a positive spatial correlation of carbon emissions; if the value is less than 0, it means there is a negative spatial correlation of carbon emissions; if the value is 0, it means there is no spatial autocorrelation of carbon emissions. The spatial weight matrix (W) is selected as the adjacency matrix since the research object is at the district and county levels. The corresponding element is 1 if two regions are adjacent and 0 otherwise.

### Spatial econometric model

To explore the spatial relationship between land use types on carbon emissions, we first construct an ordinary least squares (OLS) model without considering spatial effects, as shown in Eq. (3):

3 $${\text{CE}}_{{{\text{it}}}} = \upalpha + \upbeta {\text{L}}_{{{\text{it}}}} + \upmu_{{\text{i}}} + \uptau_{{\text{i}}} + \varepsilon_{{{\text{it}}}}$$

where ${\mathrm{CE}}_{\mathrm{it}}$ is carbon emissions, $\mathrm{\alpha }$ is a constant term, ${\mathrm{L}}_{\mathrm{it}}$ is the core explanatory variable, i.e., the area of land use type, $\upbeta$ is the coefficient of land use type, ${\upmu }_{\mathrm{i}}$ is an individual fixed effect, ${\uptau }_{\mathrm{i}}$ is a time-fixed effect, and ${\upvarepsilon }_{\mathrm{it}}$ is an error term.

Suppose the spatial autocorrelation test proves the existence of the spatial correlation effect of carbon emissions. In this case, we will construct a spatial econometric model to analyze the spatial spillover effect of land use type and carbon emissions in greater detail. The spatial econometric model includes the spatial lag model (SAR), spatial error model (SEM), and spatial Durbin model (SDM). SDM contains the spatially lagged terms of the independent and dependent variables, which avoids the impact of the coefficient estimation of the descriptive and error terms caused by the omission of variables, as shown in Eq. (4)^40^:

4 $${\text{CE}}_{{{\text{it}}}} = \updelta \sum\limits_{{{\text{t}} = 1}}^{{\text{N}}} {{\text{W}}_{{{\text{it}}}} {\text{CE}}_{{{\text{it}}}} } + \upalpha + \upbeta {\text{L}}_{{{\text{it}}}} + \varphi \sum\limits_{{{\text{t}} = 1}}^{{\text{N}}} {{\text{W}}_{{{\text{it}}}} {\text{L}}_{{{\text{it}}}} } + \upmu_{{\text{i}}} + \uptau_{{\text{t}}} + \varepsilon_{{{\text{it}}}}$$

where $\mathrm{it}$ is the year $\mathrm{t}$ of the $\mathrm{i}$ th district and county in Hangzhou (i = 1, 2,…13; t = 2000, 2005, 2010,…2020), $\mathrm{\alpha }$ is a constant term, $\upbeta$ is the regression coefficient of the land use type area, ${\varphi }$ denotes the spatial regression coefficient of the land use type area, ${\mathrm{W}}_{\mathrm{it}}$ is the spatial weight matrix between district i and district j, ${\upmu }_{\mathrm{i}}$ denotes the regional fixed effect, ${\uptau }_{\mathrm{t}}$ denotes the time fixed effect, and ${\varepsilon }_{\mathrm{it}}$ denotes the random error term.

## Results

### Evolution of land use and carbon emissions in Hangzhou

**Figure 3:**
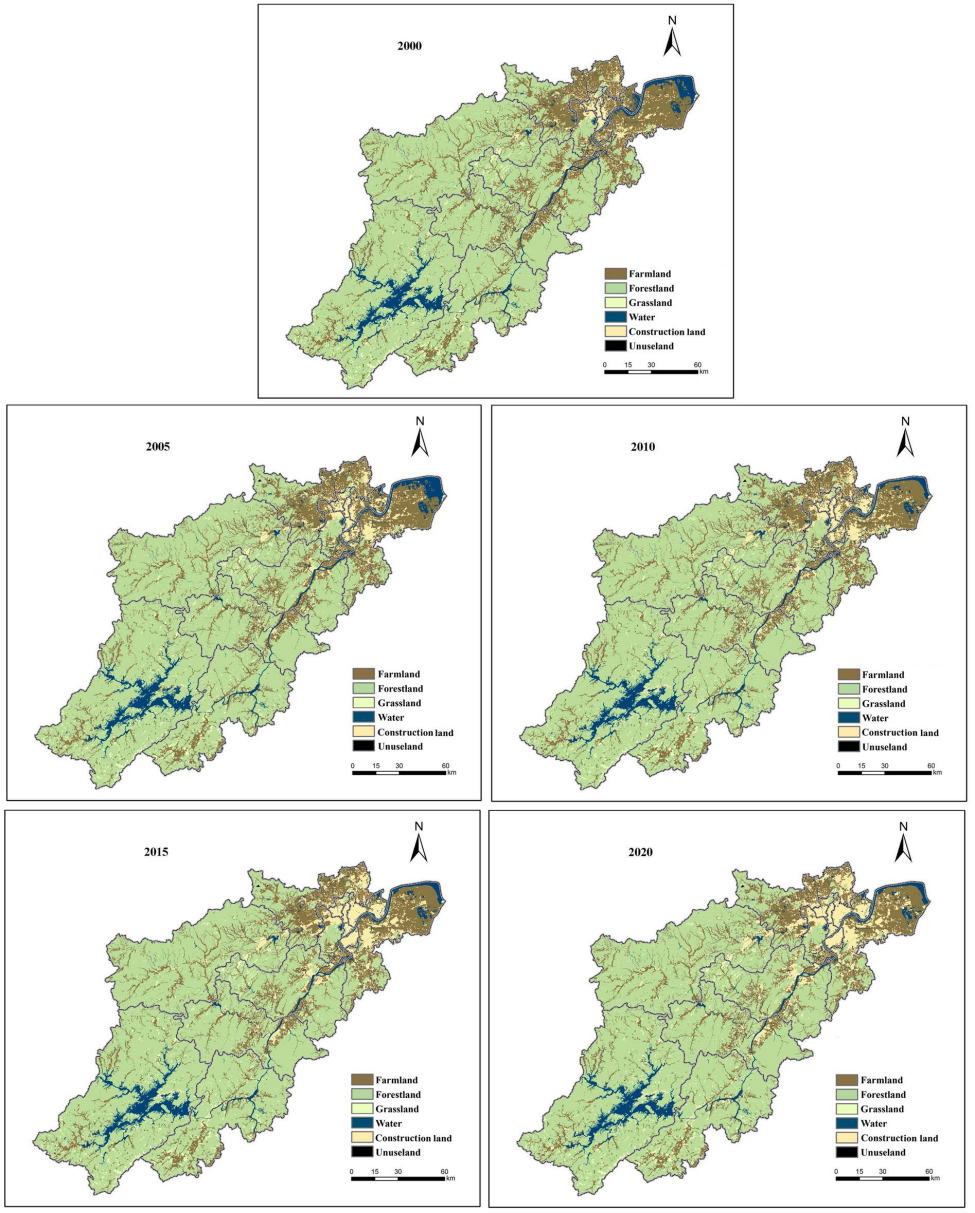
Land use in Hangzhou from 2000 to 2020 (using Arc GIS 10.8. Source of vector maps: http://datav.aliyun.com/portal/school/atlas/area_selector. Source of land use data: http://www.resdc.cn).

**Figure 4:**
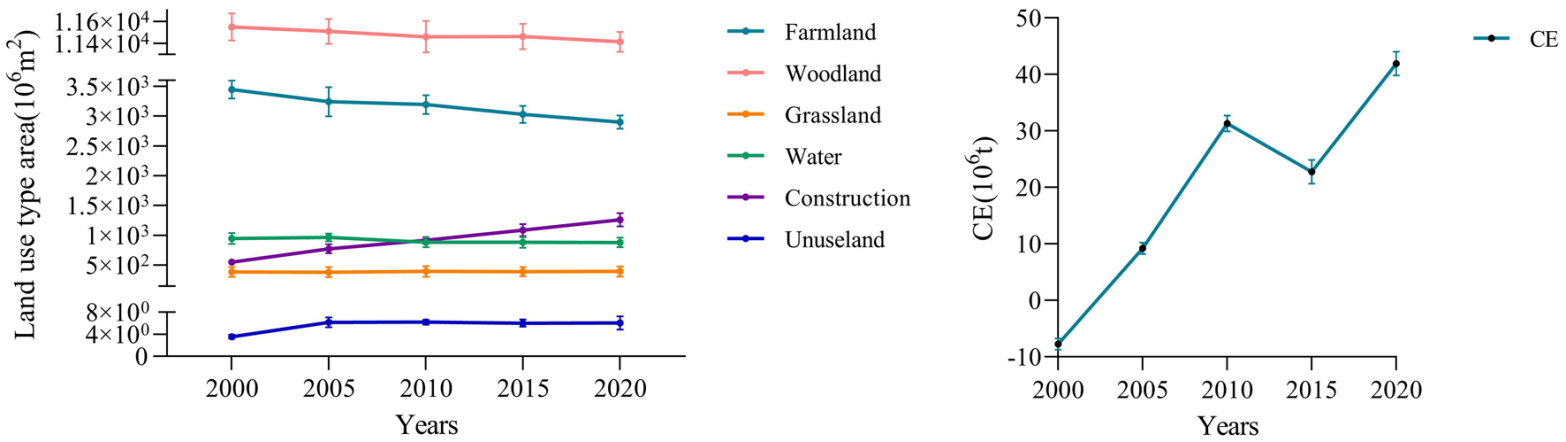
Trends in changes in land use area and carbon emissions from 2000 to 2020.

As depicted in Figs. 3 and 4, woodland accounts for more than 65% of the total land area in Hangzhou, followed by arable land, which accounts for more than 17% of the area. In contrast, construction land, water area, and grassland proportions are only 7%, 5%, and 2%, respectively. In terms of distribution, arable land, construction land, and waters are primarily located in the eastern region, decreasing from east to west; woodland, grassland, and unused land are mainly located in the western region, increasing from east to west. Regarding area change, Hangzhou's land use is dominated by improved construction land and decreased water and arable land. Grassland, construction land, and unused land increased by 1.94%, 129.66%, and 69.89% between 2000 and 2020, while arable land, woodland, and water area decreased by 15.83%, 1.17%, and 6.9%, respectively. Since unused land accounts for less than 1% of Hangzhou's total area, the study's results on underused land are insignificant, and unused land is therefore not included in the study model.

As shown in Fig. 4, Hangzhou produced a total of 38.245 million tons of carbon emissions from 2000 to 2020, and the carbon emissions in Hangzhou increased approximately 5.5013 times between 2000 and 2020, which reflects the socio-economic development of Hangzhou over the past 20 years, which has led to the increase in carbon emissions. From different periods, the carbon emission of Hangzhou in 2000 was − 8.4542 million tons, indicating that carbon storage was more significant than carbon production. In 2005 carbon emissions reached 9.9222 million tons, and the carbon emission in this period not only turned damaging to positive, but the carbon source was much larger than the carbon storage. Comparing 2010 to 2005, carbon emissions increased approximately 2.2596 times, reflecting the accelerated urban development that Hangzhou experienced from 2000 to 2010, resulting in carbon sources that were significantly larger than carbon stocks. By 2015, carbon emissions had decreased from 32.3427 tons in 2010 to 24.2856 tons in 2015. However, between 2015 and 2020, carbon emissions are projected to increase to 38.245 million tons, a new 20-year high.

### Spatial autocorrelation test

The global Moran's I tested the spatial autocorrelation of carbon emissions in Hangzhou (Table 1). The Moran's I value from 2000 to 2020 were all significant at the 1% level and positive, indicating that carbon emissions have a positive spatial auto-correlation in Hangzhou. However, the Moran index progressively decreases from 0.509 to 0.145 between 2000 and 2020, showing a continuous decline in spatial agglomeration.

**Table 1:** The Moran’s I index.

Years	Moran’s I
2000s	0.509***
2005s	0.359***
2010s	0.213***
2015s	0.219***
2020s	0.145***

The symbols in the table indicate ***p < 0.01.

**Figure 5:**
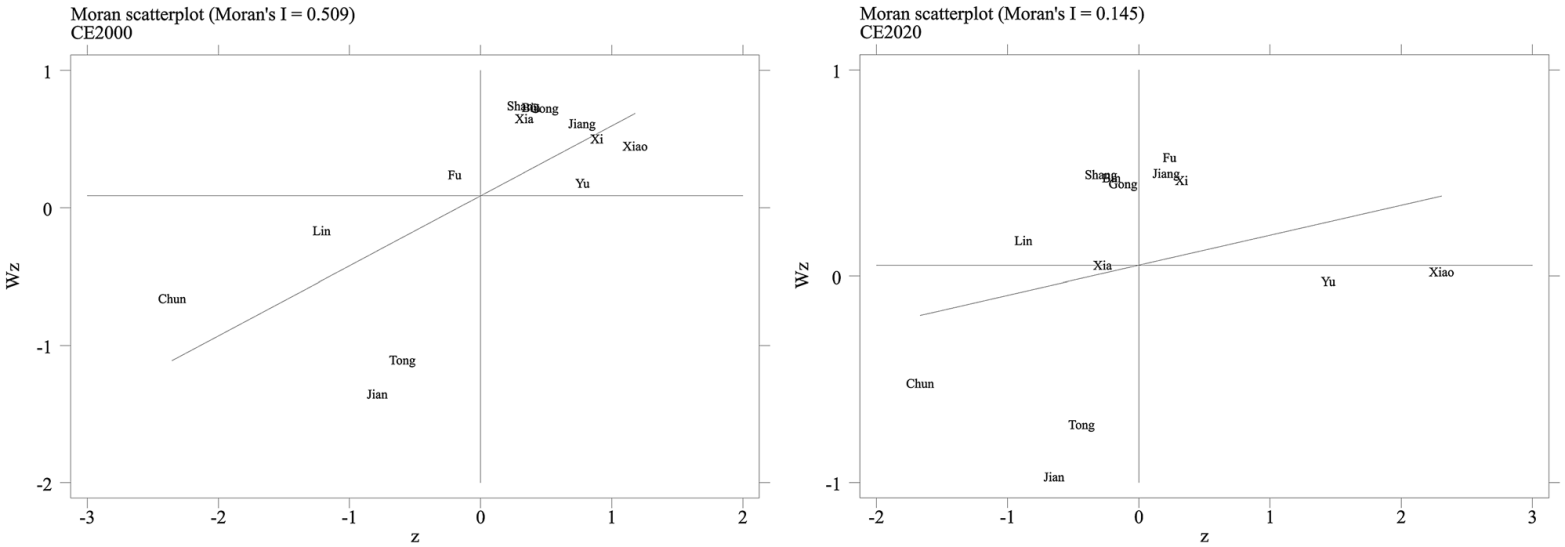
Moran scatter chart of carbon emissions from 2000 to 2020.

**Figure 6:**
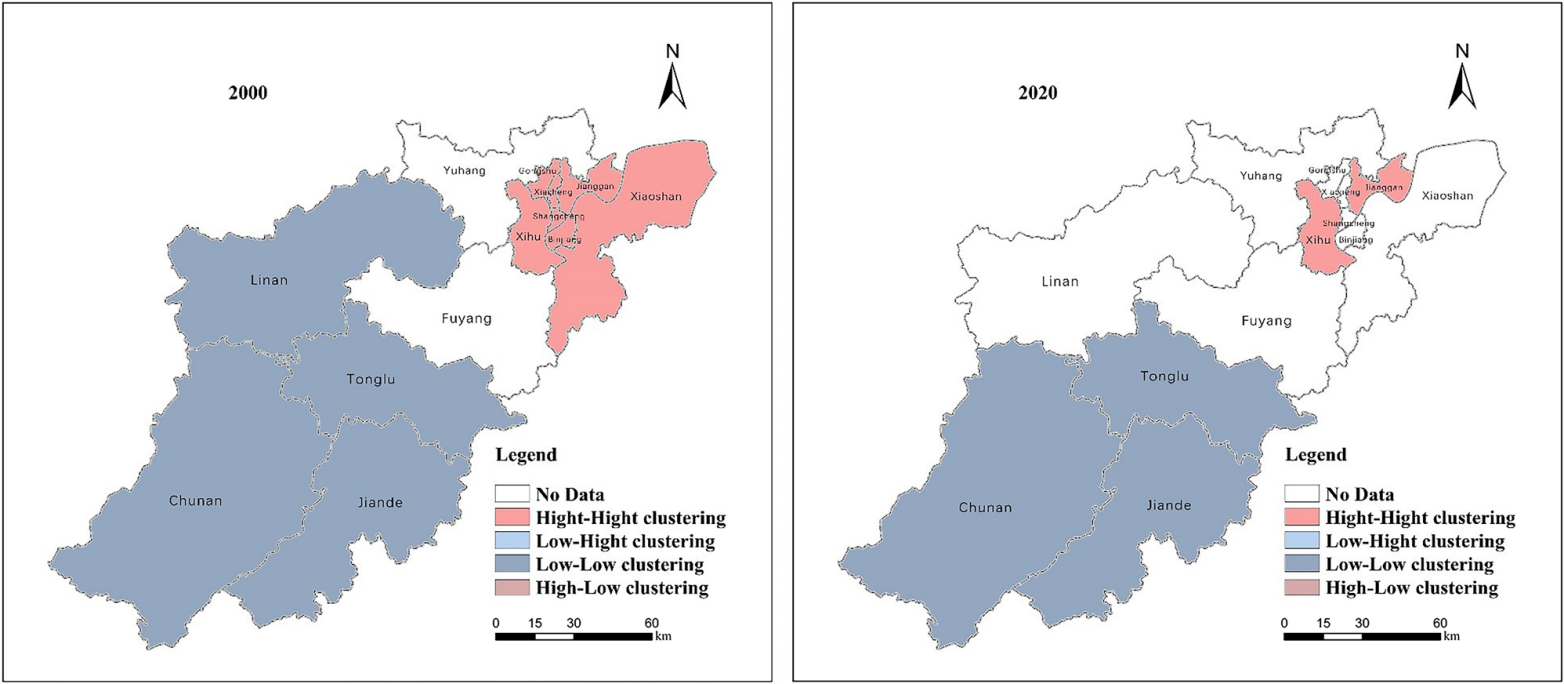
Spatial aggregation distribution of carbon emissions from 2000 to 2020 (using Arc GIS 10.8 and GeoDa. Source of vector maps: http://datav.aliyun.com/portal/school/atlas/area_selector).

We analyze the specific geographic spatial aggregation by local Moran's I to reflect regional carbon emission differences. According to the scatter distribution in Fig. 5, carbon emissions are mainly clustered in the first and third quadrants and gradually move to the second quadrant, indicating that the spatial distribution of carbon emissions in Hangzhou is in the form of "high-high" clustering and "low-low" clustering. Combining with Fig. 6, we find that the "high-high" aggregation is mainly distributed in the eastern part of Hangzhou, while the western part shows the "low-low" aggregation. Specifically, in 2000, the spatial distribution of carbon emissions in the part of the west of Hangzhou, i.e., Lin'an District, Chun'an County, Tonglu County, and Jiande City, was a "low-low" agglomeration, while the spatial distribution of carbon emissions in the eastern part, i.e., Shangcheng District, Xiacheng District, Gongshu District, Jianggan District, Xihu District, Binjiang District, and Xiaoshan District, was a "high-high" agglomeration. Lin'an District, Shangcheng District, Xiaocheng District, Gongshu District, and Binjiang District will be to the second quadrant by 2020, while Xiaoshan District will be to the fourth quadrant. The spatial autocorrelation of carbon emissions in Hangzhou demonstrates that local factors and factors influence a region's carbon emissions in neighboring regions.

### OLS results

As shown in Table 2, we analyze the impacts of various land types on carbon emissions without considering spatial effects using OLS. The R^2^ value is 0.952, and the significance of the F-test indicates a strong model fit and stability. According to the results, the arable and grassland regions failed the 10% significance test. The influence of other land types is, in descending order, construction land (6.3482), water area (− 0.3352), and woodland (− 0.2573). Every 1% expansion of construction land area will increase carbon emissions by 6.3482%; the increase of water area and woodland area can effectively reduce carbon emissions, and every 1% increase of water area and the woodland area will reduce carbon emissions by 0.3352% and 0.2573%, respectively, with the influence degree of water area slightly more extensive than that of the woodland area.

**Table 2:** OLS result.

Variables	Coeff
Farmland	− 0.0014
Forestland	− 0.2573***
Grassland	0.7088
Water	− 0.3352*
Construction land	6.3482***
Constant	− 6988.966***
R^2^	0.952
F-test	218.82
Prob > F	0.0000

The symbols in the table indicate 0.05< *p < 0.1, ***p < 0.01.

### Result of the spatial model

In the OLS results, most land types significantly affect carbon emissions, combined with the spatial autocorrelation test confirming that carbon emissions are spatially autocorrelated, so it is necessary to include spatial effects for analysis. Before analyzing the spatial effect, selecting an appropriate spatial econometric model is necessary. We examine this using the LR, Wald, and Hausman tests. As shown in Table 3, both the LR and Wald tests are significant at 1% level, indicating that the model rejects the degradation of SDM to SAR and SEM. The Hausman and effect tests are significant at 1% and 5%, respectively, indicating SDM's applicability of mixed effects. Fageda^41^ pointed out that when the trend of multiple explanatory variables is non-significant or unaltered, the detection of fixed effects may be imprecise and random effects should be used instead. Because some districts and counties lack forests and grasslands, we compared the Log-likelihood values of the fixed effect and random effect outcomes. The Log-likelihood value of the random effect was − 690.4103, whereas the fixed effect was − 658.2872; therefore, the random effect model was more applicable to this study.

**Table 3:** Model inspection.

Inspection type	Testing statistics
LR_lag_	7.71***
LR_error_	7.45***
Wald_lag_	8.32***
Wald_error_	7.85***
Hausman	43.28***
Time	23.65***
Ind	54.77***

The symbols in the table indicate ***p < 0.01.

### Spatial Dubin model results

As shown in Table 4, after considering spatial effects, the R^2^ and log-likelihood values of SDM are greater than OLS, indicating that SDM is more applicable than OLS. Therefore, it is appropriate to include spatial effects when analyzing the relationship between land use types and carbon emissions. In addition, the spatial lag term ρ is significantly positive at the 10% level, indicating that land use change has a positive spatial spillover effect on carbon emissions, i.e., an increase in regional carbon emissions drives the growth of carbon emissions in the surrounding neighboring areas.

**Table 4:** Decomposition of spatial spillover effect.

Land use type	Main	Wx
Farmland	0.5258**	0.8868**
Woodland	− 0.6562***	− 1.4395***
Grassland	7.0753***	− 2.8601**
Water	− 2.9796***	− 0.4889
Construction land	15.9394***	38.9922***
rho	0.2774**	
Log-likelihood	− 690.4103	
R^2^	0.9631	

The symbols in the table indicate 0.01< **p < 0.05, ***p < 0.01.

Regarding the influence effect, all land use types pass the significance test. The expansion of cultivated land area (0.5258), grassland area (7.0753), and construction land area (15.9394) can promote carbon emissions, among which the influence of construction land area is much more significant than that of cultivated land and grassland, indicating that the expansion of construction land area is the main reason for the increase of carbon emissions in Hangzhou. In addition, the area of woodland (− 0.6562) and the area of water (− 2.9796) have a suppressive effect on carbon emissions, with the influence degree of the area of water is significantly greater than that of woodland, indicating that the area of water is the most significant factor in reducing carbon emissions.

In terms of overflow effects, all land types except watershed are significant at the 5% and 1% levels, indicating that expanding the region's watershed area can only reduce carbon emissions. Arable land (0.8868) and construction land area (38.9922) have positive spatial spillover effects, indicating that the area of these two land types can increase the carbon emissions of the neighboring districts and counties. Woodland (− 1.4395) and grassland (− 2.8601) have a negative spatial spillover effect, indicating that the expansion of these two lands can reduce carbon emissions in neighboring districts and counties.

### Spatial spillover effect

We decomposed them to analyze further the spatial spillover effects of land use types on carbon emissions (Table 5). The direct effect reflects the effect of land use changes on carbon emissions in the region. All land use types are significant at the 5% level. The area of cultivated land, grassland, and construction land have positive direct effects on carbon emissions with coefficients of 0.6268, 7.0223, and 20.1548, respectively, indicating that the expansion of the area of cultivated land, grassland, and construction land in the region has a contributing effect on carbon emissions in the region, with the area of construction land having a much more significant impact than that of cultivated land and grassland. In addition, woodland and water area have a negative direct effect on carbon emissions, with influence coefficients of − 0.8073 and − 3.1801, indicating that the expansion of woodland and water area in the region will reduce carbon emissions in the region, with the woodland area having a more significant influence degree than the water area.

**Table 5:** Decomposition of spatial spillover effects.

Land use type	Direct effect	Indirect effect	Total effect
Farmland	0.6268**	1.4805**	2.1073**
Woodland	− 0.8073***	− 2.3337***	− 3.141***
Grassland	7.0223***	− 1.2478	5.7745***
Water	− 3.1801***	− 2.0254	− 5.2055
Construction land	20.1548***	62.7348**	82.8896***

The symbols in the table indicate 0.01< **p < 0.05, ***p < 0.01.

The indirect effect reflects the impact of neighboring land use changes on carbon emissions in the region. Grassland and watershed fail the 10% significance test, whereas cropland, forestland, and construction land all pass at the 5% significance level. The area of cultivated land and construction land have significant positive indirect effects on carbon emissions, with impact coefficients of 1.4805 and 62.7348, respectively, indicating that each 1% expansion of cultivated land and construction land area in the surrounding area will increase carbon emissions in the region by 1.4805% and 62.7348%, respectively. The woodland area has a significant negative indirect effect on carbon emissions with a coefficient of − 2.3337, indicating that each 1% expansion of the woodland area in the surrounding area will reduce carbon emissions in the region by 2.3337%. It indicates that the area of construction land is the primary cause of the rise in regional carbon emissions and the immediate land type for the rise in interregional carbon emissions. However, the woodland area is less effective than a watershed in reducing carbon emissions in the region. Regarding the spillover effect, the woodland is the only land type that can reduce inter-regional carbon emissions.

## Discussion

### Spatial distribution of carbon emissions in Hangzhou

Through the empirical study, we found that the carbon emissions in Hangzhou have a significant spatial correlation, mainly clustered in the eastern and western areas of Hangzhou. Among them, the east region shows the aggregation of high carbon emissions, and the region of the west shows the aggregation of low carbon emissions. This finding is consistent with the studies of Xia et al.^42^ Wang et al.^43^ The main reason is that the land use in the eastern region is mainly transformed into construction land. The active economic activities affect the carbon sequestration provided by other land types. With the change in economic development, financial investments are gradually withdrawn from some eastern regions, so the aggregation of high carbon emissions is decreasing yearly. The primary land use type in the western region is woodland and water environment. The west region has shown the carbon sink over the carbon source during the study period. However, due to the input of economic activities, the construction land area has progressively increased, resulting in the disparity between the carbon sink and carbon source gradually narrowing, and the circumstance of low carbon emission aggregation has changed.

### The impact of land use types on carbon emissions

We find that construction land is the only land type that can increase carbon emissions, woodland and watershed can decrease carbon emissions, and neither the negative effect of cropland nor the positive effect of grassland is statistically significant. The direction of the influence of construction land, woodland, and water area did not change after the spatial effect was included, and both cropland and grassland exhibited significant positive effects. When the analysis was conducted in individual regions, the carbon sequestration effect of cropland and grassland was counterbalanced by carbon emissions from agriculture-related energy consumption and human activities. Under the influence of production, trade, and competition, agricultural economic cooperation did not advance inter-regional carbon reduction. It became an essential carbon source in cities due to the spatial effect. Grasslands are also subject to interregional exploitation of related resources, and their contribution to carbon emissions is significantly more significant than that of cultivated land. Regarding the results of grasslands, studies by relevant researchers concluded that grasslands are an essential type of carbon sink land for cities^14–16^, which contradicts the findings of this study. This discrepancy is likely because these studies did not account for the influence of spatial effects. There are also substantial discrepancies in the distribution of grasslands between study regions, which may also influence the results.

After including land use types in the spatial effect analysis, the spatial lag term is significantly positive, indicating that land use changes in Hangzhou have a positive spatial spillover effect on carbon emissions. The results of SDM indicate that the increase in carbon emissions in Hangzhou is primarily due to the increase in construction land area. On the one hand, the expansion of construction land area promotes the agglomeration of population, transportation, and industry, which increases energy demand and, consequently, carbon emissions^44^. On the other hand, expanding the construction land area will reduce the area of other land categories, thereby influencing the carbon sequestration efficacy of natural ecosystems^45^. In addition, the area of cropland and grassland contributes to carbon emissions, with the effect of the grassland area being significantly more significant than that of the cropland area. The reason may be that the distribution of grassland is near construction land, which, under the influence of human activities, saturates the carbon sequestration efficacy of grassland and makes it an important carbon source for cities^46,47^. Under the influence of the agricultural economy, the expansion of arable land area reflects changes in agricultural production techniques, investments, and management patterns, which increase energy consumption and inhibit the carbon sequestration of crops themselves^48,49^. Moreover, expanding arable land affects other land types with carbon sequestration, thereby decreasing the carbon stock of ecosystems^50,51^. The increase in area facilitates carbon sequestration by plants and microorganisms through respiration, which allows woodland and water areas to reduce carbon emissions effectively. Water exhibits a more substantial carbon sequestration effect than forest land, possibly due to Hangzhou's more robust ecological protection policies for the water environment than forests. Under strict protection policies, human activities are limited in developing and utilizing water bodies. It is important to note that our study cannot imply that woodlands are less effective at carbon sequestration than watersheds based on the land area because the factors determining carbon sequestration capacity include biomass and species density others^52,53^.

The direct effect results were almost consistent with the SDM correlation. The indirect effect reflects the spatial spillover effect of land use type on carbon emissions. The positive spatial spillover effect of arable land area and construction land area on carbon emissions indicates that expanding arable land area and construction land area will promote carbon emissions in the region and the surrounding neighboring areas. This may be due to crucial economic trade activities and competitive relationships among districts and counties, resulting in carbon emission linkages^17,36,54^. Only forests have a negative spatial spillover effect on carbon emissions, and the degree of impact is far greater than the direct effect, indicating that the inhibitory effect of forests on carbon emissions in Hangzhou is mainly reflected in the spillover effect. Expanding the area is conducive to cross-regional carbon sinks and reducing carbon emissions^28,55^. Although the negative spillover effect of forests is smaller than the direct effect of water underwater ecological protection policies, the spillover effect of water bodies on carbon emissions is insignificant. This is owing to the unequal distribution of water between districts and counties, which makes water incapable of providing cross-regional carbon sequestration.

### Suggestions for land use optimization

In regional urban planning, land structure optimization are the primary means to improve the carbon balance between human activities and ecosystems. To achieve low-carbon development in Hangzhou, the spatial effect of carbon emissions should be entirely played, and the collaborative carbon emission management mechanism should be promoted. On the one hand, the management of carbon source land use types and the ecological protection of carbon sink land types need to be strengthened; on the other hand, the spatial spillover effect of land use types on carbon emissions should be given full play, and the optimization of land use structure should drive the neighboring areas to reduce carbon emissions. For the spatial differences of carbon emissions in Hangzhou, the western region needs to optimize the protection of the current land use, and the eastern region of Hangzhou needs to improve the land use structure.

For the western part of Hangzhou, woodland and the water are relatively concentrated and can provide carbon sequestration benefits under strict ecological protection. In this regard, it is necessary to further collaborate with neighboring districts and counties to construct environmental protection policies, reduce the resource and economic development of waters and woodland should be diminished, and make woodland and waters interconnected and coordinated as much as possible when land planning to strengthen the negative spillover effect on carbon emissions in neighboring areas.

For the eastern portion of Hangzhou, the relative concentration of construction land and arable land reflects the promotion of urban economy and agricultural economic development on the overall carbon emission, which requires not only adjusting the direction of related economic growth but also limiting the disorderly expansion of construction land and arable land into waters and woodland. For arable land types, planning must minimize the impact on other vegetation-covered lands and the economy, strengthening inter-regional agricultural economic development and cooperation, promoting agricultural production processes' transformation to modern low-carbon production technologies, and realizing the synergistic effect of low-carbon agriculture. For construction land, it is necessary to encourage the development of low-carbon production technology and the connection between high- and low-economic development districts and counties. Concurrently, it is necessary to limit the spread to other land types and prevent the positive spatial spillover effect of construction land expansion on the carbon emissions of neighboring districts and counties. In land use planning, we should focus on the uniform distribution of various types of land, reduce resource development and increase the types of grassland and woodland primarily for ecological protection, distinguish the functions of environmental protection, resource development, and urban living, reduce resource use and economic development of the natural environment by formulating relevant policies, form a synergistic ecological governance system, and improve the position of the land. Urban parks with ecological functions are enhanced and expanded to improve urban carbon sequestration benefits, mitigate the urban heat island effect, and indirectly reduce energy consumption.

### Limitations and future prospects

This study examines the influence mechanism and spatial spillover effects of land use categories on carbon emissions from the perspective of spatial effects. It proposes optimization suggestions for land planning in Hangzhou based on research results. During the research process, this study has limitations and potential for future optimization.

Existing datasets were used in the statistics of urban carbon emissions, and it is difficult to avoid errors among different datasets. The limitation of the study is that it is reasonable to calculate and analyze the carbon stocks and sources of land use types using top-down or bottom-up approaches, taking into account the regional climatic and geographical conditions.

This study analyzed the effect of land use types on carbon emissions by considering only spatial factors. Future studies should employ a spatiotemporal weight matrix that combines spatial and temporal dimensions to analyze spatiotemporal effects to investigate the relationship between the two in depth.

## Conclusions

Reducing carbon dioxide emissions is the primary strategy for combating climate change and a prerequisite for China's sustainable development. As an essential factor influencing the carbon sequestration efficacy of ecosystems, land use significantly affects the achievement of "carbon neutrality." This study analyzes the spatial spillover effect of land use type area on carbon emissions in every district and county of Hangzhou from 2000 to 2020. The following are our conclusions:

From 2000 to 2020, the changes in land types in Hangzhou show an increase in construction land and grassland and a decrease in the watershed, woodland, and arable land area. During the study period, with the economic development of Hangzhou, the area of grassland increased by 1.9358%, the area of construction land increased by 1.2966 times, and the areas of the watershed, woodland, and arable land decreased by 18.8925%, 1.1706%, and 15.8312%, respectively. 38.025 million tons of carbon emissions were generated in Hangzhou during the 20 years, with significant positive spatial clustering characteristics. Due to the difference in economic development, the eastern region shows a "high-high" agglomeration, and the western region shows a "low-low" agglomeration. With the economic transformation in the east region and the economic development in the region of the web, the spatial agglomeration effect gradually decreases.

Land use change has a significant spatial lag in carbon emissions, and the spatial spillover effect of carbon emissions in Hangzhou under land use change is positive. Energy consumption continues to increase under the influence of urban economic development and agricultural production demand, and the expansion of arable land and construction land not only has a catalytic effect on carbon emissions but also drives neigh-boring districts and counties to produce carbon emissions under the influence of economic trade and competition among districts and counties. Under the influence of human activities, the carbon sequestration efficiency of grassland is suppressed, resulting in the promotion of carbon emissions from grassland. In contrast, increasing grassland area through inter-regional resource development promotes carbon emissions from neighboring districts and counties. The carbon sequestration efficiency of woodlands and watersheds can effectively reduce urban carbon emissions. Due to the uneven distribution of watersheds, they do not significantly affect carbon emissions in the neighboring districts and counties. However, the expansion of woodland areas can increase the carbon storage in the surrounding neighboring counties and thus has a negative spatial spillover effect on carbon emissions.

## Data Availability

The datasets used or analyzed during the current study are available from the corresponding author on reasonable request.
